# Dynamics of Dental Education, Organization, and Regulation of Dental Practice in Europe 2016-2023

**DOI:** 10.1016/j.identj.2025.100852

**Published:** 2025-06-10

**Authors:** Thomas Gerhard Wolf, Peter Lingström, Jacques Deniaud, Ralf Friedrich Wagner, Gerhard Konrad Seeberger, Oliver Zeyer, Alfred Büttner, Angela Rovera, Paula Perlea, Simona Dianišková, Freddie Sloth-Lisbjerg, Guglielmo Campus

**Affiliations:** a Department of Cariology, Institute of Odontology, Sahlgrenska Academy, University of Gothenburg, Gothenburg, Sweden; b Department of Restorative, Preventive and Pediatric Dentistry, School of Dental Medicine, University of Bern, Bern, Switzerland; c Department of Periodontology and Operative Dentistry, University Medical Center of the Johannes Gutenberg University Mainz, Germany; d Free Association of German Dentists FVDZ (Freier Verband Deutscher Zahnärzte), Bonn, Germany; e French Dental Association ADF (Association Dentaire Française), Paris, France; f National Association of Statutory Health Insurance Dentists KZBV (Kassenzahnärztliche Bundesvereinigung), Cologne, Germany; g Italian Dental Association AIO (Associazione Italiana Odotoiatri), Torino, Italy; h Swiss Dental Association SSO, Bern, Switzerland; i German Dental Association (BZÄK) (Bundeszahnärztekammer), Berlin, Germany; j Italian Dental Association ANDI (Associazione Nazionale Dentisti Italiani), Roma, Italy; k Department of Endodontics, Carol Davila University of Medicine and Pharmacy, Bucharest, Romania; l Department of Orthodontics, Medical Faculty, Slovak Medical University, Bratislava, Slovakia; m Council of European Dentists CED, Brussels, Belgium; n Danish Dental Association TF (Tandlægeforeningen), Copenhagen, Denmark; o Department of Oral and Maxillofacial Sciences, Sapienza University of Rome, Rome, Italy

**Keywords:** Corporate dentistry, Dental education, Dental practice, Europe, Group practices, Investor, Legal framework, Oral healthcare center, Regulations

## Abstract

**Background:**

This study examines shifts in dental education, organization of dentists, changes in regulation of dental practice across European countries and comparing differences between 2016 and 2023 of member states of the FDI World Dental Federation and WHO-Europe region.

**Methods:**

Surveys conducted by the ERO-FDI in 2016 and 2023 included 45 countries (34 ERO and 11 non-members). Data on practice types, legal frameworks, education, and organization were collected via national dental associations. Statistical analyses employed t-tests and Fisher's exact tests to compare the two surveys over time.

**Results:**

Private practice (self-employment) remained the dominant model (48.65%±28.28%, confidence interval (CI) [43.11 / 54.19]), followed by employment in private practice (24.32% ± 20.33%, CI [20.34/28.30]) and group practice (15.27%±20.39%, CI [11.27/19.27]), public health system (13.76%±20.17% (CI [9.81, 17.71]), municipal/national clinic (8.98%±17.86% CI [5.48/12.48]), oral healthcare center (6.61%±14.19% CI [3.83/9.39]), university clinic (4.90%±6.82% CI [3.56/6.24]), and industry (0.36%±0.78% CI [0.21/0.51]). Statistically significant growth was observed in group practice (two-tailed; F=14.53 *P <* .01) and oral healthcare center (two-tailed; F=30.72 *P <* .01). Male/female dental student ratio remained stable at approximately 1:2 (two-tailed; F=0.87, *P =* .66 (m); F=0.85, *P =* .60 (f)). A total of two-thirds of the countries allow non-dental investor-led oral healthcare centers (*P* = 1.00).

**Conclusions:**

European dentistry is currently undergoing significant changes, including an increasing adoption of corporate and group practice models, approximately a 2:1 female to male ratio in dental education, and a growing urban-rural divide in care. Legal frameworks and the increasing involvement of non-dental investors could affect the quality and accessibility of care, particularly in rural areas. Future research should examine the long-term impact of these changes on patient care, dentist satisfaction, and the demand for flexible working models.

## Introduction

Over recent decades, the organization of dental practice in Europe has undergone significant changes. Independent practices have been increasingly replaced by group practices and investor-backed structures, driven by legislative reforms, economic factors, and a new generation of dentists less inclined toward traditional self-employment.^1^, ^2^, ^3^, ^4^, ^5^, ^6^ Health care market liberalization has further commercialized dentistry in many European countries, accompanied by an increase in the number of dentists, driven partly by the establishment of private universities and expanded training programs.^1^^,^^2^ Additionally, dentistry has been shaped by globalization, technological advancements, digitalization, and sustainability.^1^, ^2^, ^3^ New practice models, such as group practices and oral healthcare centers, have emerged.^6^ However, these developments have widened access to dental services but raised concerns about care quality and unequal distribution between urban and rural areas.^5^ The legal and structural framework conditions for dental care differ considerably in Europe, which has a direct impact on access to care.^6^ While the European Commission regularly seeks harmonization within the EU, health policy remains a national matter. Health plays only a supplementary role within the EU under Article 168 of the Treaty on the Functioning of the EU, with limited responsibilities except in areas such as pharmaceuticals or medical devices.^6^^,^^8^ Directive 2005/36/EC sets minimum standards for the training of dentists, which ensure automatic recognition of qualifications.^8^^,^^9^ Meanwhile, the increasing commercialization of dentistry, driven by oral healthcare centers set up by non-dental investors and municipalities, is exacerbating inequalities in urban care.^10^

At the same time, there are reports of serious regional differences in dental care. In some countries, such as the United Kingdom, there is a significant shortage of skilled workers despite a high number of registered dentists, which considerably limits access to dental services.^11^ In Ireland and other countries, there is an oversupply of practices in urban areas, while rural regions are underserved.^12^ Across Europe, the number of graduates from dental programs has increased over the last 20 years, but it is questionable whether the graduates actually work with patients in dental practices or whether they work in other areas, such as public health, industry, or research.^1^^,^^3^ When it comes to the keyword ‘work-life balance’, which is frequently mentioned by young dentists today, the expected working life time must be taken into account when calculating the supply of the population, since nowadays, for example, part-time working models have become more attractive.^1^^,^^3^^,^^7^^,^^13^ It can be observed that an increasing number of older dentists are still working in their dental practices, which, especially in rural areas, are finding it difficult to recruit young dentists willing to take over their practice. This is a major challenge that has been exacerbated by the COVID-19 pandemic,^14^, ^15^, ^16^ although pilot projects are already in place in various rural areas that are attempting to attract young colleagues to future rural practice locations while they are still studying.^17^

The study aims to systematically analyze changes in the dental education, organization of dentists, and regulation of dental practices in the member states of the European Regional Organization (ERO) of the FDI World Dental Federation and the WHO European Region from 2016 to 2023 to identify key trends and differences, to better understand and support the future design of oral healthcare in Europe.

## Material and methods

### Study design and data collection

This comparative study is based on two surveys conducted by the European Regional Organization of the FDI World Dental Federation (ERO).^3^^,^^7^ Both surveys examined the organization and regulatory framework of oral healthcare centers and dental education as well as modern forms of professional practice in ERO member countries. The first survey was conducted between December 2015 and April 2016 and the questionnaire was developed by the ERO Working Group “Liberal Dental Practice”.^3^ The questionnaire was distributed to the contact persons of the national dental associations in 37 ERO member countries. The second survey, conducted between December 2022 and April 2023, was aimed to re-examine the organizational and regulatory frameworks of dental practices and dental education, as well as modern forms of professional practice in ERO member countries.^7^ The same validated questionnaire^3^ was sent to 34 ERO member countries and 11 non-ERO member countries in the WHO European Region (Figure). The Lime Survey platform (Lime Survey GmbH, Hamburg, Germany) was used to distribute and collect responses. To ensure the highest possible response rate, participants were reminded twice by the ERO Secretariat. The working environment of dentists was categorized into distinct groups: private practice (self-employment), employed in private practice, group practice, oral healthcare centers, municipal/national clinic, university clinic, public health services, industry, and others. The dentist supply rate of each country was calculated using data from the World Bank (2021) to account for population size.^18^

**Fig:**
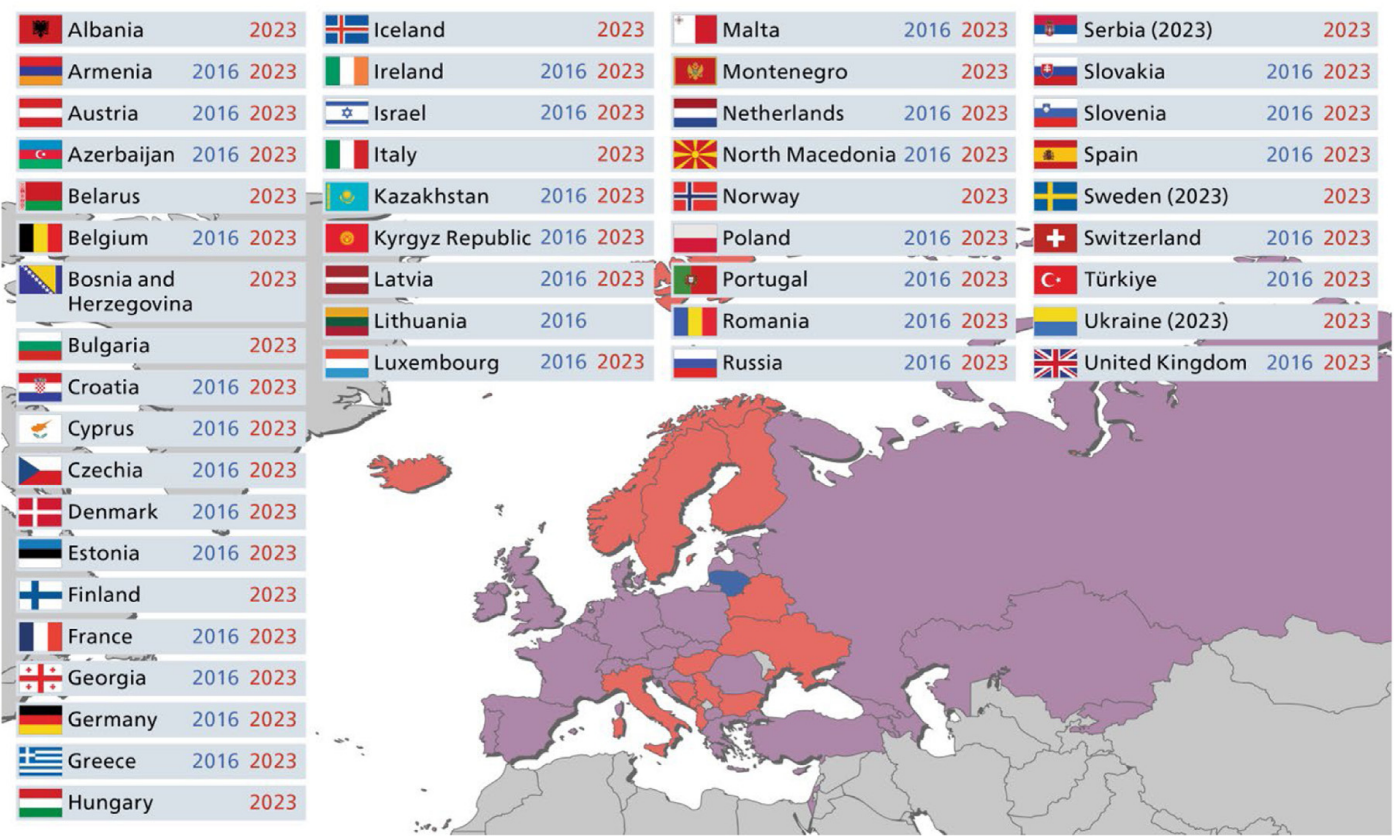
Countries of the WHO European region participating in the studies in 2016 and 2023. The participating countries are color-coded according to their participation in each respective year (countries in blue: 2016 only, red: 2023 only, purple: 2016 and 2023).

### Statistical analysis

All data collected from the surveys were coded and entered an Excel™ spreadsheet (Microsoft Corp., Redmond, WA, USA) for analysis. For statistical analysis, both mean comparisons and the exact Fisher test were used to examine differences between the two survey years, 2016 and 2023.^3^^,^^7^ The mean values and standard deviations of the various professional work environments of dentists (*e.g.*, self-employed with their own practice, employed in a practice, in dental care centers, municipal clinics, or industry) were compared using one-tailed and two-tailed t-tests. In addition, an F-test for testing the variances was performed to analyze differences in the dispersion of the data. The Fisher exact test was used to examine the distribution of various groups authorized to establish a dental care center. This analysis was carried out for groups such as non-dental investors (without dental or medical training), dentists, physicians, local communities, health insurance companies, and companies. Statistical analysis was performed using the software OpenEpi (www.openepi.com; last accessed 8 May 2025), an open-source software for epidemiological calculations in public health.

## Results

The results were summarized in four main areas: the rate of dental care provision and the number of graduates (Table 1), the professional working environments of dentists (Table 2), the legal requirements for dental practice (Table 3), and the conditions for setting up an oral healthcare center (Table 4).

**Table 1:** Comparison of number of dentists in the country and dental care rate (inhabitants per dentist), number of graduates, state and private universities, and sex distribution of dental students.

	2016 Dentists in the country (n) *Mean ±*	2023 Dentists in the country (n) *Mean ±*	2016 Dental care rate (Inhabitants per dentist) (n) *Mean ±*	2023 Dental care rate (Inhabitants per dentist) (n) *Mean ±*	2016 Graduates (n) *Mean ±*	2023 Graduates (n) *Mean ±*	2016 State Universities (n) *Mean ±*	2023 State Universities (n) *Mean ±*	2016 Private Universities (n) *Mean ±*	2023 Private Universities (n) *Mean ±*	2016 Males (%) *Mean ±*	2023 Males (%) *Mean ±*	2016 Females (%) *Mean ±*	2023 Females (%) *Mean ±*
Countries participating only 2016	14083.82 ± 18894.93	15906.94 ± 19624.81	1692.55 ± 1141.40	1469.56 ± 872.25	684.19 ± 1240.92	701.42 ± 1257.53	8.71 ± 13.94	9.97 ± 17.17	1.71 ± 3.66	2.48 ± 5.75	36.11 ± 10.76	36.05 ± 12.93	63.89 ± 10.76	63.95 ± 12.93
Countries participating in both 2016 and 2023	14403.03 ± 19094.57	13210.76 ± 17579.53	1719.27 ± 1148.24	1459.79 ± 800.80	766.52 ± 1025.87	627.89 ± 913.56	8.91 ± 14.11	8.33 ± 15.00	1.76 ± 3.71	2.21 ± 5.07	36.65 ± 10.52	36.13 ± 12.06	63.35 ± 10.52	63.63 ± 11.89
One-tailed	*P =* .52		*P =* .54		*P =* .62		*P =* .52		*P =* .52		*P =* .58		*P =* .42	
Two-tailed		*P =* .53		*P =* .96		*P =* .78		*P =* .66		*P =* .83		*P =* .98		*P =* .91
F-test for variances	F=1.02 *P =* .95	F 0.80 *P =* .49	F=1.01 *P =* .97	F=0.84 *P =* .59	F=0.68 *P =* .28	F=0.53 *P =* .05	F=1.02 *P =* .94	F=0.76 *P =* .40	F=1.03 *P =* .94	F=0.78 *P =* .43	F=0.96 *P =* .90	F=0.87 *P =* .66	F=0.96 *P =* .90	F=0.85 *P =* .60

**Table 2:** Comparison of kind of work environment (%).

	Private practice (self-employed) *Mean ±*		Employed in private practice *Mean ±*		Group practice *Mean ±*		Oral healthcare center *Mean ±*		Municipal/ national clinic *Mean ±*		University clinic *Mean ±*		Public health system *Mean ±*		Industry *Mean ±*		Others *Mean ±*	
Countries participating only 2016	53.29 ± 27.37		19.02 ± 18.92		3.71 ± 5.35		2.29 ± 2.56		5.36 ± 10.23		3.83 ± 4.27		10.66 ± 27.02		0.14 ± 0.38		1.91 ± 2.93	
Countries participating in both 2016 and 2023	52.58 ± 28.98	48.65 ± 28.28	21.56 ± 20.59	24.32 ± 20.33	13.56 ± 19.04	15.27 ± 20.39	5.79 ± 12.28	6.61 ± 14.19	6.92 ± 17.54	8.98 ± 17.86	4.62 ± 6.46	4.90 ± 6.82	7.67 ± 15.06	13.76 ± 20.17	0.40 ± 0.87	0.36 ± 0.78	3.51 ± 6.23	2.78 ± 5.63
One-tailed	*P =* .46		*P =* .71		*P =* 1 .00		*P =* .97		*P =* .69		*P =* .74		*P =* .28		*P =* .96		*P =* .93	
Two-tailed		*P =* .47		*P =* .24		*P < .0*1		*P =* .05		*P =* .26		*P =* .40		*P =* .58		*P =* .10		*P =* .38
F-test for variances	F=1.12 *P =* .74	F=1.07 *P =* .86	F=1.18 *P =* .62	F=1.15 *P =* .68	F=12.67 *P <* .01	F=14.53 *P <* .01	F=23.01 *P <* .01	F=30.72 *P <* .01	F=2.94 *P <* .01	F=3.05 *P <* .01	F=2.29 *P =* .02	F=2.55 *P =* .01	F=0.31 *P <* .01	F=0.56 *P =* .07	F=5.24 *P <* .01	F=4.21 *P <* .01	F=4.52 *P <* .01	F=3.69 *P <* .01

**Table 3:** Legal requirements for the foundation of an oral healthcare center, self-reliant dental work possible immediately after graduation, residency mandatory, over-or undersupply in urban and rural areas, and changes in the ratio of the number of practices in urban and rural areas in the last 10 years (n [%]).

Legal requirements for the foundation of an oral healthcare center				
	One-tailed		Two-tailed	
	2016	2023	2016	2023
Yes	22 (78.6)	21 (80.8)	23 (79.3)	29 (80.6)
No	6 (21.4)	5 (19.2)	6 (20.7)	7 (19.4)
Total	28 (100.0)	26 (100.0)	29 (100.0)	36 (100.0)
Fisher Exact test *P =* .55			Fisher Exact test *P =* 1 .00	

Self-reliant dental work possible immediately after graduation				

	One-tailed		Two-tailed	
	2016	2023	2016	2023

Yes	21 (65.6)	20 (69.0)	22 (66.7)	26 (63.4)
No	11 (34.4)	9 (31.0)	11 (33.3)	15 (36.6)
Total	32 (100.0)	29 (100.0)	33 (100.0)	41 (100.0)
Fisher Exact test *P =* .50			Fisher Exact test *P =* .81	

Residency mandatory				

	One-tailed		Two-tailed	
	2016	2023	2016	2023

Yes	25 (92.6)	29 (90.6)	7 (29.2)	13 (36.1)
No	3 (7.4)	6 (9.4)	17 (70.8)	23 (73.9)
Total	31 (100.0)	24 (100.0)	24 (100.0)	36 (100.0)
Fisher Exact test *P =* .36			Fisher Exact test *P =* .78	

Over- or undersupply in urban region				

	One-tailed		Two-tailed	
	2016	2023	2016	2023

Yes	14 (53.8)	22 (82.1)	15 (55.6)	32 (82.1)
No	12 (46.2)	5 (17.9)	12 (44.4)	7 (17.9)
Total	26 (100.0)	27 (100.0)	27 (100.0)	39 (100.0)
Fisher Exact test *P =* .03			Fisher Exact test *P =* .03	

Over- or undersupply in rural region				

	One-tailed		Two-tailed	
	2016	2023	2016	2023

Yes	19 (79.2)	20 (71.4)	20 (80.0)	31 (77.5)
No	5 (20.8)	8 (28.6)	5 (20.0)	9 (22.5)
Total	24 (100.0)	28 (100.0)	25 (100.0)	40 (100.0)
Fisher Exact test *P =* .38			Fisher Exact test *P =* 1 .00	

Changes in the ratio of the number of practices in urban and rural areas in the last 10 years				

	One-tailed		Two-tailed	
	2016	2023	2016	2023

Yes	18 (75.0)	12 (56.7)	18 (72.0)	17 (56.7)
No	6 (25.0)	9 (43.3)	7 (28.0)	13 (43.3)
Total	24 (100.0)	21 (100.0)	25 (100.0)	30 (100.0)
Fisher Exact test *P =* .17			Fisher Exact test *P =* .27

**Table 4:** Comparison of who may establish an oral healthcare center (n [%]).

Investors without a dental or medical education				
	One-tailed		Two-tailed	
	2016	2023	2016	2023
Yes	25 (89.3)	22 (84.6)	26 (89.7)	29 (82.9)
No	3 (10.7)	4 (15.4)	3 (10.3)	6 (17.1)
Fisher Exact test *P =* .46			Fisher Exact test *P =* .49	

Dentists				
	One-tailed		Two-tailed	
	2016	2023	2016	2023

Yes	27 (96.4)	24 (96.0)	28 (96.6)	33 (97.1)
No	1 (3.6)	1 (4.0)	1 (3.4)	1 (2.9)
Fisher Exact test *P =* .73			Fisher Exact test *P =* 1 .00	

Medical doctors				
	One-tailed		Two-tailed	
	2016	2023	2016	2023

Yes	25 (92.6)	20 (97.0)	26 (92.9)	29 (90.6)
No	2 (7.4)	3 (13.0)	2 (7.1)	3 (9.4)
Fisher Exact test *P =* .42			Fisher Exact test *P =* 1 .00	

Local municipalities				
	One-tailed		Two-tailed	
	2016	2023	2016	2023

Yes	22 (88.0)	21 (87.5)	23 (88.5)	28 (84.9)
No	3 (12.0)	3 (12.5)	3 (11.5)	5 (15.1)
Fisher Exact test *P =* .64			Fisher Exact test *P =* .72	

Health insurances				
	One-tailed		Two-tailed	
	2016	2023	2016	2023

Yes	19 (76.0)	15 (68.2)	19 (73.1)	21 (65.6)
No	6 (24.0)	7 (31.8)	7 (26.9)	11 (34.4)
Fisher Exact test *P =* .39			Fisher Exact test *P =* .58	

Business companies				
	One-tailed		Two-tailed	
	2016	2023	2016	2023

Yes	20 (80.0)	16 (76.2)	21 (80.8)	22 (75.9)
No	5 (20.0)	5 (23.8)	5 (19.2)	7 (24.1)
Fisher Exact test *P =* .52			Fisher Exact test *P =* .75	

Others				
	One-tailed		Two-tailed	
	2016	2023	2016	2023

Yes	13 (76.5)	4 (57.1)	13 (76.5)	6 (60.0)
No	4 (23.5)	3 (42.9)	4 (23.5)	4 (40.0)
Fisher Exact test *P =* .32			Fisher Exact test *P =* .42	

Legal regulation for establishment of an oral healthcare center				
	One-tailed		Two-tailed	
	2016	2023	2016	2023

Yes	21 (80.8)	22 (84.6)	22 (81.5)	30 (83.3)
No	5 (19.2)	4 (15.4)	5 (18.5)	6 (16.7)
Fisher Exact test *P =* .50			Fisher Exact test *P =* 1 .00	

### Dental workforce and education

While the number of dentists in the 2016 participating countries increased from 14,403.03 ± 19,094.57 to 15,906.94 ± 19,624.81 (two-tailed; F=0-80 *P =* 49), the dental care rate (inhabitants per dentist) decreased from 1692.55 ± 1141.40 to 1469.56 ± 872.25, which was not statistically significant (two-tailed; F=0.84 *P =* .59) (Table 1). The number of graduates in dentistry (two-tailed; F=0.53 *P =* .05) and the number of state (two-tailed; F=0.76 *P =* .40) and private universities (two-tailed; F=0.78 *P =* .43) also showed slight, albeit statistically insignificant, fluctuations, with a slight increase in the number of state and private universities. The sex distribution of dental students remained constant, with women continuing to make up the majority at around 64% (two-tailed; F=0.85 *P =* .60) and men around 36% (two-tailed; F=0.87 *P =* .66), with no statistically significant changes over time (Table 1).

### Practice settings and organizational structures

Group practices in countries that participated in both surveys had a significantly higher mean (two-tailed; F=14.53 *P <* .01) (Table 2). Statistically significant differences in variability were also found for oral healthcare centers (two-tailed; F=30.72 *P <* .01). The public health system (two-tailed; F=0.56 *P =* .07), university clinics (two-tailed; F=2.55 *P <* .01), industry (two-tailed; F=4.21 *P <* .01), municipal/national clinics (two-tailed, F=3.05 *P <* .01), and others (two-tailed F=3.96 *P <* .01) indicated significant differences, suggesting a greater diversity of these practice settings in the countries that participated in both surveys in 2016 and 2023 (Table 2). No statistically significant changes were detected in the categories private practice (self-employed) (53.29±27.37 in 2016 *vs* 48.65±28.28 in 2023; two-tailed F=1.07 *P =* .86) and employed in an office (19.02±18.92 in 2016 *vs*. 24.32±20.33 in 2023; two-tailed F=1.15 *P =* .68). These results demonstrate a significant diversification of the professional environment for dentists, particularly with the expansion of group practices, oral healthcare centers, and industry involvement. At the same time, stability in categories such as private practices (self-employed) and employed in an office suggests enduring traditional structures in certain areas of the profession.

### Regulatory framework for dental practice

Table 3 compares the legal requirements for setting up an oral healthcare center, private practice (self-employed) immediately after graduation, mandatory vocational training after graduation, over- or under-supply in urban or rural areas, and a change in the number of practice structures in urban and rural areas over the last 10 years between 2016 and 2023. The legal provisions for establishing oral healthcare centers were consistently reported by most participating countries, with no significant differences in 78.6% of countries in 2016 and 80.8% in 2023 (*P =* .55, *P =* 1 .00). Similarly, about two-thirds of the participating countries reported that it is possible to become a self-employed dentist immediately after graduation (65.6% in 2016 vs. 69.0% in 2023), with no significant change over time (*P =* .50, *P =* .81). Most countries in both years indicated that there was a mandatory vocational training (92.6% in 2016 compared to 90.6% in 2023), with no significant differences (*P =* .36, *P =* .78). However, in urban areas, the perception of oversupply or undersupply increased significantly from 53.8% in 2016 to 82.1% in 2023 (*P =* .03), while perception in rural areas remained stable (79.2% in 2016 vs. 71.4% in 2023; *P =* .38, *P =* 1 .00). Finally, reports of changes in the ratio of practices between urban and rural areas decreased slightly, with no statistically significant differences (*P =* .17, *P =* .27). These results show stability in most categories, except for a significant increase in perceived oversupply or undersupply in urban areas.

### Authorization to establish oral healthcare centers

Table 4 compares the groups that were entitled to establish an oral healthcare center between 2016 and 2023. The majority are still legally allowed to establish oral health centers, including dentists (96.4% in 2016 vs. 96.0% in 2023) and physicians (92.6% in 2016 vs. 97.0% in 2023). Investors without dental or medical training (non-dental) had the right to establish oral health centers in 89.3% of states in 2016 and 84.6% in 2023, while local municipalities had the right to establish oral health centers in 88.0% of states in 2016 and 87.5% in 2023. A slight decrease was seen for other stakeholders, such as health insurance organizations (76.0% in 2016 vs. 68.2% in 2023) and businesses (80.0% in 2016 vs. 76.2% in 2023). The “others” category fell significantly from 76.5% in 2016 to 57.1% in 2023, although this difference was not statistically significant (*P* > .05). However, no statistically significant differences were found between 2016 and 2023, indicating that the legal situation for establishing an oral health center remained unchanged and stable in most countries.

## Discussion

The present study was designed and carried out to examine the changes in the education, organization, and regulatory framework in the European Regional Organization (ERO) of the FDI World Dental Federation and WHO-Europe region over a period of seven years.

### Commercialization and investor-driven practice structures

The changes observed in this study towards cooperative practice forms are consistent with developments described in the current literature. Earlier work shows similar developments, suggesting that dental practices worldwide are increasingly being financed by investors without a dental background (non-dental), raising questions about the quality of patient care and the ethical principles of professional practice.^1^^,^^4^^,^^10^ This trend, which can be seen in Europe and other regions, reflects an increasing commercialization in the health sector, especially in dentistry. In some countries, non-dental investors can still open oral healthcare centers.^5^ Self-employed dentists in smaller practices are concerned about this development and are of the opinion that these financial non-dental investors and private equity entrepreneurs, who are not familiar with the provision of care and whose primary goal is profit, endanger both the liberal profession and patient-centered care.^10^ In the EU member states, various features have been established to characterize liberal professions, such as “the public interest of the service, the professionally and economically independent performance of tasks, the self-employed and personal provision of services, the existence of a special relationship of trust between client and contractor, and the subordination of the interest of maximizing profits”.^19^, ^20^, ^21^ In this context, the increasing gap in provision between urban and rural areas is also frequently discussed, as investors tend to locate their oral healthcare centers in the commuter belts of large cities and in affluent areas, while rural areas are increasingly underserved but often unattractive to profit-oriented investors due to lower profit expectations. Even though no significant influence of dentist density as a measure of competition intensity on overtreatment recommendations has been found so far, the phenomenon of overtreatment is still not negligible, even though it is known that dentists with shorter waiting times are more likely to suggest unnecessary treatments.^22^ In addition, there is the fact that in some countries it has been observed that foreign-investor-supported oral healthcare centers primarily aim for private services, while vulnerable groups such as children or the disabled are hardly treated or not treated at all due to the high costs and lower financial coverage of the services.^10^

### Urban-rural disparities in dental care provision

One key finding from the presented data is the increase in oral healthcare centers and cooperative practice structures such as group practices. In addition, an increasing gap between dental care in urban and rural areas was identified. It is known so far that urban regions are increasingly oversupplied, while rural areas remain undersupplied in many European countries.^5^ The increase in private universities and the feminization of the profession are further important findings, with the proportion of female dental students known to have risen to approximately two-thirds in recent years.^19^ Studies also show that dentists prefer to open practices in urban areas due to the better infrastructure and higher dentist density, while rural areas are increasingly being underserved, even though there is a trend towards more doctors setting up their own practices.^23^, ^24^, ^25^ Although financial incentives bring short-term improvements, it can be observed that in the long term only structural changes, such as a better work-life balance, can sustainably promote the settlement of dentists in rural areas.^26^

### Developments in dental education

The study results on dental education document the growing proportion of private universities in countries such as Spain or Türkiye.^7^ The proportion of female dental students, which now almost two-thirds, reflects the “feminization” of dentistry, which is also emphasized in global studies.^19^ This requires gender-equitable measures and more flexible working models to meet the specific needs of women in dentistry and of the future generation of dentists in general.^13^^,^^19^^,^^27^

### Limitations

The high response rate of the two studies examined is a major strength of the study and emphasizes the importance of the need for nation states to collect such data. The numerous countries participating in the study enable representative coverage of ERO member countries and provide a solid basis for the analysis in the European region.^7^ The broad geographical coverage, with a total of 46 countries included in both surveys, also allows for detailed insights into the European region and nearby countries examined. By including regulatory and organizational frameworks, the study also provides a comprehensive overview of dental practice and its relevant implications, such as mandatory residency after graduation.^6^ However, one potential weakness of the study is its dependence on national dental organizations for data provision. This data may be incomplete, outdated, or even influenced to produce a certain result, which, in the sum of possible distortions, could affect the validity of the results. In addition, the study focuses mainly on quantitative data and does not consider any qualitative aspects, such as the satisfaction of dentists or the quality of dental education. These are important factors that should be given more consideration in future studies to obtain a more complete picture of dental care. Another weakness is the insufficient data basis for new openings and closures of dental practices. This information would be important to better understand the dynamics of market changes and their impact on dental care. In total, results of this study can be considered representative for the ERO zone and the WHO European Region, supported by the broad geographical coverage and high response rate of the questionnaires. Nevertheless, it is difficult to transfer the results to other regions outside Europe. The forms of dental practice and educational structures are strongly dependent on the specific social, cultural and economic conditions of the countries.^6^ In countries with completely different health systems, such as the USA or emerging markets, completely different changes, emerging trends or challenges could arise.^28^ Another factor that complicates the transferability of results is the different regulatory frameworks in ERO countries. In some countries, non-dental investors are allowed to establish oral healthcare centers, while in others this is more strictly regulated. This can affect both the competitiveness of dental practice structures and the quality of patient care.^4^^,^^7^

## Conclusions

The findings of this study highlight changes and challenges in the dental profession that require attention. The increasing prevalence of cooperative practice forms such as group practices and oral healthcare centers offers notable advantages, including specialization and shared responsibility, which should be further encouraged. However, the growing disparity in access to dental care between urban and rural areas underscores the urgent need for targeted further study and, if necessary, political action to improve conditions in the affected regions. Moreover, the demands of young dentists for flexible working models emphasize the necessity of supporting work-life balance and, considering the current student ratio of almost two thirds female students, promoting women in leadership positions. The increasing commercialization of dentistry through the involvement of non-dental investors is giving rise to concerns about the potential impact on the quality of care and access to care, as well as a pure focus on economic interests that are diametrically opposed to the nature of a liberal profession. However, this phenomenon warrants further investigation to ensure patient outcomes remain the priority. Finally, future research should adopt a more comprehensive approach by incorporating qualitative dimensions such as the satisfaction of dentists and patient perspectives to provide a holistic understanding of the evolving dental landscape. These insights are crucial for shaping sustainable and equitable dental care systems in the future.

## Funding

This work was supported by the European Regional Organization (ERO) of the FDI World Dental Federation.

## Author contributions

*T.G.W., P.L., and G.C.:* Project administration, Supervision, Data curation, Methodology, Formal analysis, Investigation, Writing− original draft, Writing− review & editing. *J.D., R.F.W., G.K.S., O.Z., A.B., A.R., P.P., S.D., and F.S.L.:* Data curation, Formal analysis, Investigation, Writing− review and editing.

## Conflict of interest

None disclosed.
